# An Alternative Approach to Investigate Biofilm in Medical Devices: A Feasibility Study

**DOI:** 10.3390/ijerph14121587

**Published:** 2017-12-17

**Authors:** Tiziana Petrachi, Elisa Resca, Maria Serena Piccinno, Francesco Biagi, Valentina Strusi, Massimo Dominici, Elena Veronesi

**Affiliations:** 1 Science and Technology Park for Medicine, via 29 Maggio 6, 41037 Mirandola, Modena, Italy; tiziana.petrachi@tpm.bio (T.P.); elisa.resca@tpm.bio (E.R.); mariaserena.piccinno@tpm.bio (M.S.P.); valentina.strusi@tpm.bio (V.S.); massimo.dominici@unimore.it (M.D.); 2 Department of Medical and Surgical Sciences for Children & Adults, University Hospital of Modena and Reggio Emilia, via del Pozzo 71, 41124 Modena, Italy; 3 B. Braun Avitum Italy S.p.A., via XXV Luglio 11, 41037 Mirandola, Modena, Italy; francesco.biagi@bbraun.com

**Keywords:** medical device, biofilm, crystal violet, bacteria, stereomicroscopy

## Abstract

Biofilms are assemblages of bacterial cells irreversibly associated with a surface where moisture is present. In particular, they retain a relevant impact on public health since through biofilms bacteria are able to survive and populate biomedical devices causing severe nosocomial infections that are generally resistant to antimicrobial agents. Therefore, controlling biofilm formation is a mandatory feature during medical device manufacturing and during their use. In this study, combining a crystal violet staining together with advanced stereomicroscopy, we report an alternative rapid protocol for both qualitative and semi-quantitative biofilm determination having high specificity, high repeatability, and low variability. The suggested approach represents a reliable and versatile method to detect, monitor, and measure biofilm colonization by an easy, more affordable, and reproducible method.

## 1. Introduction

Biofilms are organized layers of Gram-positive and Gram-negative bacteria attaching to both abiotic and biotic surfaces [1,2]. They can colonize metal surface of medical devices such as heart valves, pacemakers, and catheters [3] adversely affecting their functions and causing severe nosocomial infections [4]. According to a report by the Italian Institute of Health, most infections are caused by biofilms formation [5]. Examples of biofilm-associated medical conditions are indwelling devices, dental plaque, upper respiratory tract infections, peritonitis, and urogenital infections often associated with an increased resistance to antimicrobial agents [6]. Antimicrobial resistance has a relevant impact on healthcare since infected individuals require longer hospitalization often associated with a poor prognosis [7]. Chemical agents or biocides can be used to sterilize metal surfaces. However, these preventive treatments are not sufficient to control biofilm formations [8]. Bacteria growing in biofilms are difficult to eradicate due to a combination of a protective sheets and for the intrinsic antibiotic resistance of involved strains [9]. There is a wide range of tests that are currently used for biofilm detection such as DNA based methods (PCR, DNA sequencing), imaging (confocal scanning laser microscopy with molecular probes, Fluorescence In Situ Hybridization (FISH) or labelled antibodies-electron microscopy) and others [10,11,12]. However, these methods can show several limitations. First, most biofilms on medical devices are due to a polymicrobial contamination, thus tests on a limited range of bacterial strains may not be sufficient [13]. Furthermore, there are still no biomarkers able to specifically identify a growing mixed bacteria population. A possibility could be the detachment of biofilms from the biomaterial for further profiling, however this may prevent the identification of the peculiar geometry and shape that can facilitate bacteria proliferation and biofilm formation. Moreover, some detection methods require large and expensive laboratory equipment and several days or weeks to perform the analyses [14]. 

Today, medical device manufacturers are more frequently involved in finding more reliable techniques for detecting, measuring, and controlling biofilm formation. The aim of this study is to propose a predictive method to screen biofilms on new materials during biomedical device manufacturing and testing. This alternative ex vivo platform might discriminate the “bio-adhesive properties” of different materials based on the presence/absence of a staining, allowing qualitative and semi-quantitative read-outs to be then combined with additional molecular and in vivo investigations. This new platform could be advantageous for manufacturers in order to perform a large-scale screening of materials and during the R&D (Research and Development) phase, before selecting the most suitable material. Moreover, this method could find space within a hospital environment for early biofilm detection.

## 2. Materials and Methods

The experimental approach was based on four phases: (1) bacterial culture and biofilm preparation; (2) crystal violet staining of biofilm populated stainless steel welded heat-exchangers; (3) detection of the biofilm by stereo microscopic qualitative analysis; and (4) measurement of biofilm populating material by semi-quantitative analysis. 

Bacterial culture media and biofilm preparation. As suggested by IEC (International Electrotechnical Commission) 60601-2-16 [15], a mix of four types of bacteria strains commonly populating medical devices was introduced: Gram-positive, aerobic *Bacillus Subtilis* spores (ATCC 6633, Biogenetics S.r.l., Padova, Italy), Gram-negative, aerobic *Pseudomonas aeruginosa* (ATCC 9027, Biogenetics S.r.l.,Padova, Italy), Gram-positive, aerobic and anaerobic *Staphylococcus aureus* (ATCC 6538, Biogenetics S.r.l., Padova, Italy), Gram-positive, aerobic and anaerobic *Enterococcus hirae* (ATCC 8043, Biogenetics S.r.l., Padova, Italy). Bacterial strains were provided by the manufactures as lyophilized pellet, containing a defined bacterial charge. After re-hydration in a defined Trypticase Soy Broth (TSB) medium (Biomérieux Italia S.p.A., Firenze, Italy), bacteria were cultured until they reached a 10^7^ logarithmic growth. After amplification, 16 × 10^7^ CFU (Colony Forming Unit)/mL of each bacteria strain were inoculated in 50 mL of TSB medium at a temperature of 37 °C for 24/48 h. This bacterial suspension was then used to contaminate eight stainless steel device specimens (5.0 × 2.5 cm) for at least two weeks at temperature of 37 °C under laminar flow hood. Eight negative controls were performed with TBS alone, omitting bacteria solution. All the devices were previously sterilized in an autoclave.

Crystal violet staining of biofilm populated biomaterial. One of the most common methods to assess biofilm formation current relies on a 96 well microtiter plate assay, which usually involves a colorimetric detection of the dye removed from the previously stained biofilm [16]. Crystal violet is a basic protein dye that marks the surface negatively charged molecules (i.e., peptidoglycan) and extracellular matrix of polysaccharides [17]. In order to avoid manipulation and artefacts due to the detachment of bacteria and their biofilm, a crystal violet staining was directly applied to the surfaces of material. After bacteria contamination, biomaterial was fixed in ice methanol for 5 min at room temperature and then washed in H_2_O. A direct staining with aqueous solution (0.25% *v*/*v*) of crystal violet (V5265, Sigma Aldrich, St Luis, MO, USA) was performed for 5 min at room temperature and, after two washes in H_2_O, biomaterials were dried until the following steps. 

Detection of biofilm by stereomicroscopic analysis. Using light stereomicroscopy (stereo zoom Microscope for Large Fields AxioZoom V16 (Carl Zeiss Microscopy GmbH, Jena, Germany), Objective Plan Neo Fluar Z 1X/0.25 FWD53.1 mm a qualitative analysis of the biofilm was performed. We acquired contiguous images applying the following parameters: magnification, 11.2×; field of view, 23.0 mm; resolution, 5.0 μm; depth of field, 0.5 μm. In order to acquire the whole surface of medical devices, we used a mosaic technology in which Zeiss software ZEN Pro (Carl Zeiss Microscopy GmbH, Jena, Germany) align, fuse, and stitch all contiguous images acquired by a semi-automatized table. 

Measurement of biofilm populating material. The whole image obtained by a mosaic reconstruction (with a ZEN Pro software) was analysed with ImageJ software [18]. Crystal violet color was digitally separated using Ruifrok and Johnston’s color deconvolution algorithm implemented as an NIH (National Institute of Health)-ImageJ macro. Code to implement this algorithm was obtained from Image J plugin processes on Red/Green/Blue (RGB) images to create three 8-bit monochrome images. The first represents crystal violet (color 1) and the other two images (colors 2 and 3) are the background. This color deconvolution technique can separate combinations of two or three colors, providing those colors sufficiently different in their red, green, or blue absorption features. This deconvolution technique does not depend neither on the threshold nor is limited by the possibility of overlapping absorption spectra. Orthonormal transformation of the original RGB image and color deconvolution can be used to determine and quantify staining densities in the different areas [19,20]. Total area was calculated in square millimeters and reported as percentage of stained area. 

Statistics. All results were presented in mean ± standard error of mean (Mean ± SEM). Student’s *t*-test was used to assess the significant differences between contaminated and uncontaminated surfaces.

## 3. Results

**Figure 1:**
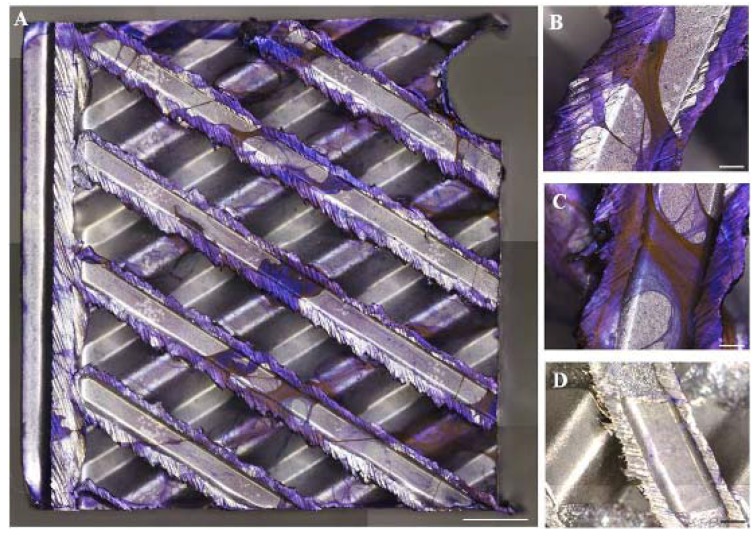
Crystal violet staining (purple) (**A**): mosaic images acquired by the motorized stereo zoom microscope AxioZoom V.16 (Zeiss). Multiple images of the large samples were merged with ZEN Pro software. Scale bars = 2000 μm (**A**) and 500 μm (**B**,**C**,**D**). Magnification = 11.2×; (**B**,**C**): magnification of a representative area of the medical devices contaminated with bacteria strains; (**D**): negative control.

Three-dimensional stereomicroscopy observations of biofilms showed the presence of a crystal violet layer of positive cells developing onto the stainless steel material surface. Subsequently, filamentous bacterial colonization occurred forming a highly complicated filamentous structure, sparsely distributed and without any preferential attachment between rough and smooth surfaces (Figure 1A–C). The specificity of staining is reported in Figure 1D showing an uncontaminated medical device as a control (Figure 1D). 

In order to monitor bacteria grow, samples of contaminated TSB were analyzed both at time 0 and after 14 days of incubation. Starting from a total amount of 64 × 10^7^ CFU/mL of four bacteria strains (16 × 10^7^ CFU/mL of each bacteria strain), after 14 days of contamination, we detected only 7.5 × 10^7^ CFU/mL in the broth, suggesting that the majority (but not the totality) of bacteria adhered to heat exchanger. 

**Figure 2:**
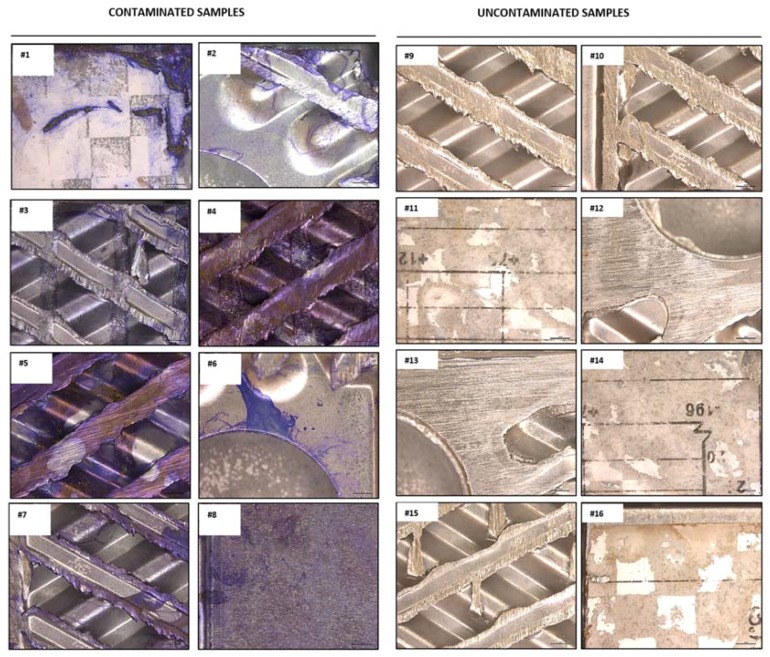
Crystal violet staining (purple) of contaminated (left panel) and uncontaminated (right panel) specimens of heat exchanger. Images were acquired by the motorized stereo zoom microscope AxioZoom V.16 (Zeiss). Scale bar = 2000 μm. Magnification = 11.2×.

All contaminated and uncontaminated analyzed specimens of heat exchanger are reported in Figure 2.

**Figure 3:**
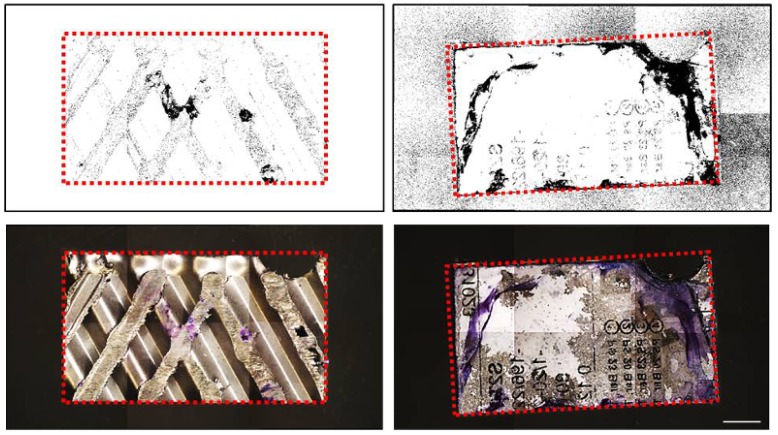
Upper panel: Mosaic images acquired by the motorized stereo zoom microscope AxioZoom V.16 (Zeiss) of two different surfaces of medical devices. Multiple images of the large samples were merged with ZEN Pro software. Lower panel: color deconvolution estimation of the original images stained with crystal violet, in purple. Dotted line shows the area of interest. Scale bar = 5000 μm; Magnification = 11.2×.

NIH ImageJ software, implemented with a color deconvolution plugin, discriminated positive channel (in purple) from background colors (Figure 3). The quantification of the total positive area was performed using a binary conversion (black/white) and reported as ratio between positive area/total area with a percentage value (Table 1). Contaminated medical devices showed a positive area of 11.87 ± 2.13% in contrast with a significantly lower signal 0.3912 ± 0.12% of the negative controls (*p* < 0.001). 

## 4. Discussion

Our analytical procedure, based on a colorimetric assay associated with microscopic and digital analyses is rapid and a relatively convenient approach able to generate qualitative and semi-quantitative data on biofilm formation on medical devices [21].

A staining method for microscopic observation of biofilms, as originally described by J. W. Arnold [16], has the advantage to rapidly monitor biofilm evolution onto physical supports. However, it does not provide a quantitative assessment of stained surface. Properly understanding and quantifying biofilm colonization represents a critical point in the development of safer and functional biomaterials. Our study, with a limitation of an in vitro investigation, combines qualitative and semi-quantitative approaches in biofilm detection and localization by advanced stereomicroscopy allowing a detailed structure–function analysis of biofilms. Moreover, mosaic technology can be applied to the analysis of large surfaces in the range of square centimeters versus classic approaches that are considering significantly smaller areas [22]. Our results, with the limitation of not providing evidence of biofilm metabolism and architecture details, suggest how this platform could be introduced during medical devices development such as during validation of antimicrobial treatments and during biomaterial design. We are also confident that the crystal violet stained area is associated with bacteria biofilm presence since the lack staining in control samples carried out by only TSB medium. 

This study allowed a simple readout to verify the presence of contamination in medical device. A further limitation of our study is the impossibility to verify the exact composition of biofilm. Crystal violet protocol associated with a washing alcohol step in the original Gram staining procedure [23] distinguishes Gram-positive from Gram-negative bacteria due to a significantly lower levels of peptidoglycans in the latter that are weakly stained by crystal violet due to the alcohol washing. In our protocol, due to the lack of alcohol, Gram-negative bacteria can still be visualized after Crystal violet staining allowing us to detect at the same time both Gram-positive and Gram-negative bacteria. 

Given that crystal violet staining is not able to identify bacteria strain as part of the biofilm, our approach could be associated by in-depth molecular assays for a precise identification of the involved strains. Further investigations shall be therefore needed in order to precisely correlate CFU counts and/or PCR results with ImageJ analysis to translate our semi-quantitative read-out into a more quantitative data. Moreover, a further optimization of our staining procedure with the reduction of washing steps could avoid biofilm detachment facilitating the sampling for additional in depth investigations. 

While our study aims also to reduce the use of in vivo investigations, we aware also that animal research is still relevant. At the same time, our method may be used to reduce these in vivo testing within a predictive platform that could be further enriched by the proposed molecular assays. 

## 5. Conclusions

The high specificity, low variability, high repeatability, and short duration of this analysis could make this protocol as a complementary method in bacteria contamination monitoring and an alternative easy, fast, and more affordable approach to detect biofilm formation. 

## Figures and Tables

**Table 1:** ImageJ quantification of the crystal violet positive area of eight specimens of contaminated heat exchanger (Samples 1–8).

Contaminated Sample	Stained Area (Pixel^2^)	mm^2^ Stained Area	% CV Positive Area	Average (%)
#1	68,469	79,801	11.2	10.388125
#2	26,411	30,782	4.15	
#3	84,321	98,276	16.13	
#4	59,147	68,936	11.21	
#5	211,735	246,777	16.11	
#6	57,876	67,455	12.135	
#7	43,758	51	6.76	
#8	35,147	40,964	5.41	
**Uncontaminated Sample**	**Stained Area (Pixel^2^)**	**mm^2^ Stained Area**	**% CV Positive Area**	**Average (%)**
#9	120	3.93	0.55	0.39125
#10	200	3.2	0.45	
#11	136	3.27	0.54	
#12	150	2.79	0.45	
#13	220	4.7	0.32	
#14	164	3.74	0.24	
#15	136	1.67	0.22	
#16	250	2.67	0.36	

Results on negative controls were obtained by omitting bacteria strains (Samples 9–16). Column 1 shows sample identity numbers. Column 2 reported total crystal violet positive area expressed in pixel^2^. Column 3 represents total crystal violet positive area expressed in mm^2^. Column 4 reports the ratio between crystal violet positive area (expressed in mm^2^) and the heat exchanger total area (expressed as percentage). CV: crystal violet.
